# ALMARVI Execution Platform: Heterogeneous Video Processing SoC Platform on FPGA

**DOI:** 10.1007/s11265-018-1424-1

**Published:** 2019-01-02

**Authors:** Joost Hoozemans, Jeroen van Straten, Timo Viitanen, Aleksi Tervo, Jiri Kadlec, Zaid Al-Ars

**Affiliations:** 1 Delft University of Technology, Delft, The Netherlands; 2 Tampere University of Technology, Tampere, Finland; 3 UTIA, Prague, Czechia

**Keywords:** ALMARVI, OpenCL, pocl, TTA, TCE, rVEX, ZYNQ

## Abstract

The proliferation of processing hardware alternatives allows developers to use various customized computing platforms to run their applications in an optimal way. However, porting application code on custom hardware requires a lot of development and porting effort. This paper describes a heterogeneous computational platform (the ALMARVI execution platform) comprising of multiple communicating processors that allow easy programmability through an interface to OpenCL. The ALMARVI platform uses processing elements based on both VLIW and Transport Triggered Architectures (*ρ*-VEX and TCE cores, respectively). It can be implemented on Zynq devices such as the ZedBoard, and supports OpenCL by means of the pocl (Portable OpenCL) project and our ALMAIF interface specification. This allows developers to execute kernels transparently on either processing elements, thereby allowing to optimize execution time with minimal design and development effort.

## Introduction

Image and video processing is an important enabler for a large number of application domains ranging from medical imaging to entertainment. These applications continue to push the requirements imposed on our computational platforms to their technological limits, both by increasing the demand for higher resolution images to be processed on the one hand, and by increasing the computational complexity of newly proposed processing algorithms on the other. As a result, applications constantly push beyond the computational capacity that general purpose computational systems can provide, and demand the design of high performance processing hardware that is customized towards the targeted video application.

Various different types of image and video processing platforms are introduced regularly to match the ever increasing requirements of the application domain. Each platform is optimized to specific types of applications, and focuses on a given set of optimization criteria. However, creating a customized processing platform is a long and costly process, both in terms of hardware design time as well as application porting time to these platforms. Some platforms provide custom made accelerators to ensure high performance, but suffer from long and expensive hardware design time [1]. Other platforms provide optimized processing cores for easy programmability, but only ensure performance for the specific targeted application [2].

In this paper, we present the ALMARVI execution platform which enables both hardware design flexibility as well as portable software deployment. The hardware architecture allows for integrating heterogeneous processing cores each optimized for their own application, thereby ensuring optimized performance. At the same time, the software stack allows for portable deployment of applications on each core based on an OpenCL programming layer [3] that has broad support in the industry for a whole range of computing platforms: multicore CPUs and GPUs, in addition to the upcoming standard of automatic hardware synthesis right from OpenCL code.

In order to facilitate integration of components (such as newly developed hardware and licensed processors and IP blocks) and to connect to cross-compatible OpenCL compilers, a new common hardware integration interface (called AlmaIF) was developed. Additionally, we present details of the implementation and integration of two heterogeneous acceleration fabrics and show how the same program implemented and compiled using a single code base can be executed on a variety of execution platforms. Software compatibility is provided by adding support for the AlmaIF interface to the Portable OpenCL environment.

This paper is organized as follows. Section 2 discusses the background and related work. Section 3 shows how the AlmaIF abstraction layer is able to virtualize away the details of the custom processing cores used in this paper. The design flows of both processors used in the prototype are discussed in Section 4, along with a detailed example on how to implement the AlmaIF interface on a processor. The hardware implementation and software support for running OpenCL programs on the platform are discussed in Section 5. We report preliminary numbers of a case study application running on the ALMARVI execution platform (an OpenCL implementation of the Sobel filter) in Section 6. Finally, Section 7 ends with the conclusions.

## Background & Related Work

### Background

Two types of processing units are used in this paper to showcase heterogeneous integration capabilities of the ALMARVI execution platform. The first supported custom processing units that supported by the platform are based on the Transport Triggered Architectures (TTA) paradigm [4]. They are generated by the TTA-based Co-design Environment (TCE), an open-source project maintained by Tampere University of Technology (TUT) [5]. These represent cores that are not just customizable regarding certain design parameters; the entire architecture of the datapath can be designed from the ground up to target an application or application domain. This includes application-specific functional units but also involves generic units such as register files, Arithmetic Logic Units (ALU), etc. These units can be configured individually (for example, register files can have different numbers of access ports - a highly influential design parameter in processor design [6]). The connectivity between each of the functional units is also a design decision. TCE generates a compiler and simulator for the processor and all of these design-time configurations can be performed using a graphical user interface.

The second processor type is the *ρ*-VEX reconfigurable VLIW processor, maintained by Delft University of Technology [7, 8]. The core has a range of design parameters that can be changed at design-time to target certain application domains. In addition, it can be reconfigured during run-time to adapt itself to the workload, making it a highly adaptive general purpose core [9]. This is realized by 1) designing a flexible connection between the processor’s datapaths and its program state storage, and subsequently 2) multiplying the state storage so that multiple programs can execute simultaneously (similar to Simultaneous Multithreading - SMT [10]). Datapaths can be assigned to program states, effectively splitting and merging the processor into one or multiple cores. They can execute a single program at high performance or multiple programs at high total throughput. The decision to split can be based on whether an application has large amounts of Instruction-Level Parallelism (ILP) or Thread-Level Parallelism (TLP).

In order to integrate these two processing units together in the same platfrom, we provide the ALMARVI Common Hardware IP Interface (AlmaIF) to enable plug’n play style of customization of the hardware platform at the system level, by allowing adding accelerators to a new hardware platform design with an easy integration to the OpenCL-based system software layer. The AlmaIF interface abstracts away implementation details so that application development only needs to focus on the OpenCL implementation. This article gives an overview of the ALMARVI execution platform used to demonstrate the capabilities of AlmaIF, which can be used for easily integrating any hardware component that supports the interface.

### Related Work

A large body of work exists that target FPGA not for prototyping purposes, but for acceleration. Prior efforts that implemented FPGA acceleration fabrics include [11], a project by Microsoft that added FPGAs to a datacenter running the Bing service. These FPGAs were equipped with programmable accelerators (softcore processors) that are highly optimized for the specific application domain. Other projects using programmable accelerators include [12] for a FFT workload and [13, 14] in the image processing application domain. These related efforts do not use OpenCL and/or heterogeneous accelerators. In [15], an exploration is performed regarding how much acceleration can be achieved using accelerators while still retaining programmability. A Heterogeneous accelerator fabric for accelerating OpenCV is presented in [16].

Within the area of FPGA accelerators, a current topic is to support high-level languages such as the one we are also using in this work, OpenCL. For example, in [17], Xilinx presents an OpenCL library of streaming acceleration units, allowing designers to quickly generate an FPGA acceleration fabric with OpenCL support. Additionally, Xilinx has introduced SdAccel, a toolset to generate hardware from OpenCL kernels [18]. An Altera equivalent is presented in [19]. In [20], a rapid prototyping framework for video processing using High-Level Synthesis is presented. RIFFA [21] is an opensource integration framework that provides an interface to connect to FPGA accelerators via PCIe. A related industry-effort aimed towards improving integration of various execution platforms is the Heterogeneous System Architecture (HSA) [22].

## AlmaIF Interface

**Figure 1:**
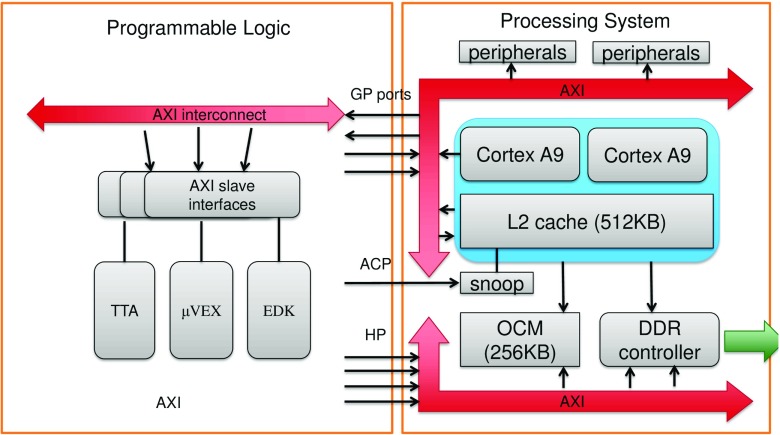
Architecture of the SoC platform.

**Figure 2:**
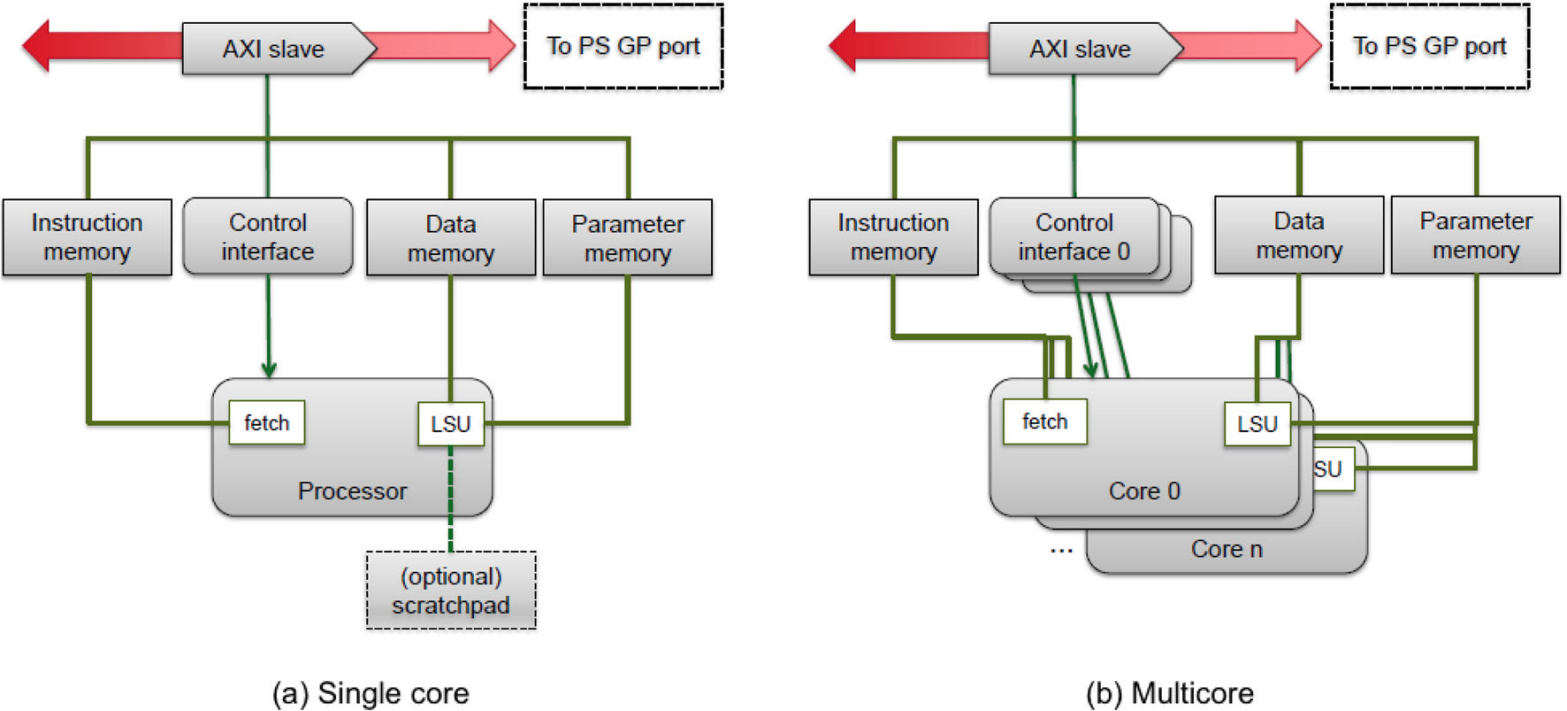
Accelerator architecture for a single core and a multicore system.

The targeted system architecture is shown in Fig. 1. The system has a single AXI interconnect, where each accelerator is connected as a slave device. A closer view of one accelerator is shown in Fig. 2a. Accelerators may also be multi-core as shown in Fig. 2b. Each core has a separate debug and control interface.

The master can access four memory sections in each accelerator:

- Control interface (CTRL). Registers used to query basic information about the accelerator and its status, and to start and stop execution. Each core in an accelerator has a separate control interface.
- Instruction memory (IMEM). Memory used to store program code for the accelerator.
- Data memory (DMEM) Memory used to store program inputs/outputs: in an OpenCL program, this would correspond to global, local and private memories.
- Parameter memory (PMEM). An additional memory used to store, e.g., OpenCL command queues.

These are mapped to the accelerator slave’s address space such that a memory address has a field long enough to store a byte address into the largest section; followed by two high bits to select between sections as shown in Table 1. The size of the AXI IP memory address can be computed as:

$$addrwidth_{IP} = 2 + max \left\{\begin{array}{l} addrwidth_{imem}\\ addrwidth_{dmem}\\ addrwidth_{pmem}\\ N_{cores} \times addrwidth_{debug} \end{array}\right. $$

The sizes can be queried through the control interface. For example, the reference TTA design has the following memory resources:

- 32KB instruction memory (4096 instructions × 8 bytes per instruction) = 15-bit address Resulting in a total address size: 2 + max(8,15,15,11) = 17, and the memory map shown in Table 2. Accesses to memory locations between sections (e.g. 0x00400) have undefined behavior. The design rationale here is that given the base address of the AXI slave, a driver program can probe memory section sizes from CTRL (which is the first memory section), and use accelerators with differently sized memories in a plug-and-play manner.

**Table 1:** Memory address regions.

00	CTRL
01	IMEM
10	DMEM
11	PMEM

**Table 2:** Example memory address regions for a single-core TTA platform.

0x00000 .. 0x003ff	CTRL
0x08000 .. 0x0ffff	IMEM
0x10000 .. 0x17fff	DMEM
0x18000 .. 0x187ff	PMEM
  The device identification part of the CTRL region allows the software layer to identify what kind of accelerator it is accessing. This is needed to determine which compiler backend is needed to compile kernels for the accelerator, or, in the case of offline compilation, which binary to use. Accelerators are identified by a device class and a device ID. The device class specifies the kind of accelerator (for instance, a TTA, a *ρ*-VEX, etc.), while the device ID should uniquely identify its configuration (for instance, in the case of a *ρ*-VEX, the issue width).
- 32KB data memory = 15-bit address
- 2KB parameter memory = 11-bit address
- Single core with a minimum-size

The capability registers contain information about the size of the memories, the number of cores within the accelerator (each core has its own copy of the CTRL region), the supported debug features (single-stepping and/or hardware breakpoints), and an AlmaIF version number. The latter allows AlmaIF to be extended later, with a backwards-compatible software layer.

The status registers supply the software layers with runtime information, such as whether the accelerator is running or not, the current program counter, and performance information. The command registers allow the software layers to reset the accelerator, to start or stop execution, to set the start address, and to set breakpoints for debugging purposes.

The instruction memory holds the kernel binary that the accelerator should execute. The data memory is intended to be used for variables local to the accelerator, which may be initialized by the host, and/or exchange of data between the accelerator and the host. The parameter memory is intended to be used as a command queue for the kernel running on the accelerator.

The CTRL memory region provides an interface according to the AlmaIF specification. Support for this interface has been implemented in the pocl runtime system, so that any accelerator that conforms to the interface can be used to run OpenCL kernels via pocl, as will be discussed in following sections.

One of the key points for the AlmaIF is to provide a hardware layer that is generic enough for sharing driver code in the OpenCL implementation layer. A hardware abstraction such as AlmaIF makes it easier to plug in different AlmaIF-compliant devices in a compute platform with only minimal changes to the driver code. This helps in making the ALMARVI execution platform composable: as devices share a common control interface, it reduces the integration effort at multiple levels due to not requiring device-specific knowledge at the simplest control functions for verification and execution purposes, for instance. The pocl runtime that utilizes AlmaIF as a hardware abstraction layer to control the execution is described in more detail in [23].

## Components

### Fully Customizable Cores: TCE

**Figure 3:**
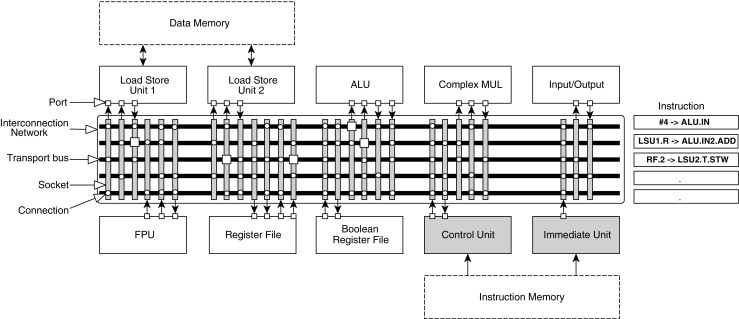
Example of a TTA datapath organization. The designer can choose the functional units, the number of transport buses (this determines the number of operations the processor can perform in one clock cycle), and whether or not the output ports of the functional units are connected to these buses.

**Figure 4:**
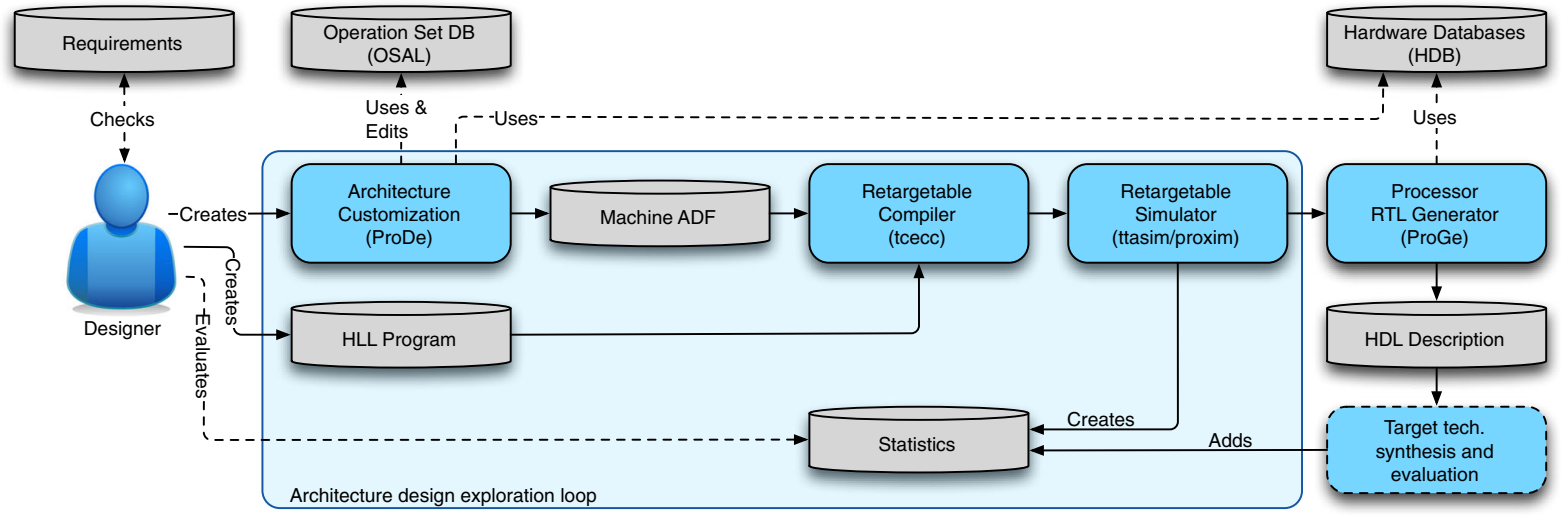
TTA accelerator design flow in TCE.

Processor customization in TCE is usually conducted as a hardware-software co-design process. The processor design is iterated by varying the processor template parameters defined using the TCE files while adapting the software to better exploit the features, such as by calling custom operation intrinsics or enhancing parallelization opportunities. An example processor design is depicted in Fig. 3. The co-design process is supported by a set of tools, as illustrated in Fig. 4. Initially, the designer has a set of requirements and goals placed to the end result. It is usual to have a real time requirement as the primary requirement, given as a maximum run time for the programs of interest and the secondary goal is to optimize the processor for low power consumption or minimal chip area. In some cases, there can be a strict area and/or power budget which cannot be exceeded, and the goal is to design as fast processor as possible that still fits below the limit. The iterative customization process starts with an initial predesigned architecture. Using a graphical user interface tool called Processor Designer (ProDe), the designer can add, modify, and remove processor components. The connectivity between the components can be customized manually using the GUI or with an automated optimizer. Each iteration of the processor is evaluated by compiling the software of interest to the architecture using TCE’s re-targetable high-level language compiler and simulating the resulting parallel assembly code using an architecture simulator. The simulator statistics give the runtime of the program and the utilization of the different data-path components, indicating bottlenecks in the design. The architecture exploration cycle enables low effort evaluation of different custom operations. For a completely new processor operation, the designer describes the operation simulation behavior in C/C++, estimates its latency in instruction cycles when implemented in hardware, and adds the operation to one of the function units in the architecture. This way it is possible to see the effects of the custom hardware to the cycle count, before deciding whether to include it in the design or not. When a design point fulfilling the requirements has been found, or more accurate statistics of a design point is needed, the designer uses a tool called Processor Generator (ProGe), which produces an RTL implementation of the processor.

#### Automatic Generation of TTA Accelerators

The main components of TCE that provide substantial automation to the design flow are:

- Processor Generator (ProGe), which produces an RTL implementation of the designed processor, and
- Design Space Explorer (explore), which can be used to perform automated transformations on TTAs.

##### RTL Generation—

Thanks to the modular TTA template, the RTL generation is straightforward and reliable; the generator utility ProGe collects component implementations from a set of hardware databases (HDBs) and generates the interconnection network which connects the units. Instruction fetch and decoding logic is generated based on the given processor architecture description file. The designer has to implement any new function units in VHDL or Verilog and add them to an HDB. A tool is available to automatically validate the function unit implementation against its architecture simulation model. The generated RTL is fed to a standard third party synthesis and simulation flow. This step generates more detailed statistics of the processor at hand, which can again drive the architectural exploration process. The detailed implementation level statistics map trivially back to the design actions at the architecture level. For example, required chip area can be reduced by removing architecture components. Similarly, adding more pipeline stages to complex function units, or reducing the connectivity of the interconnection network helps increasing the maximum clock frequency.

##### Design Space Exploration—

**Figure 5:**
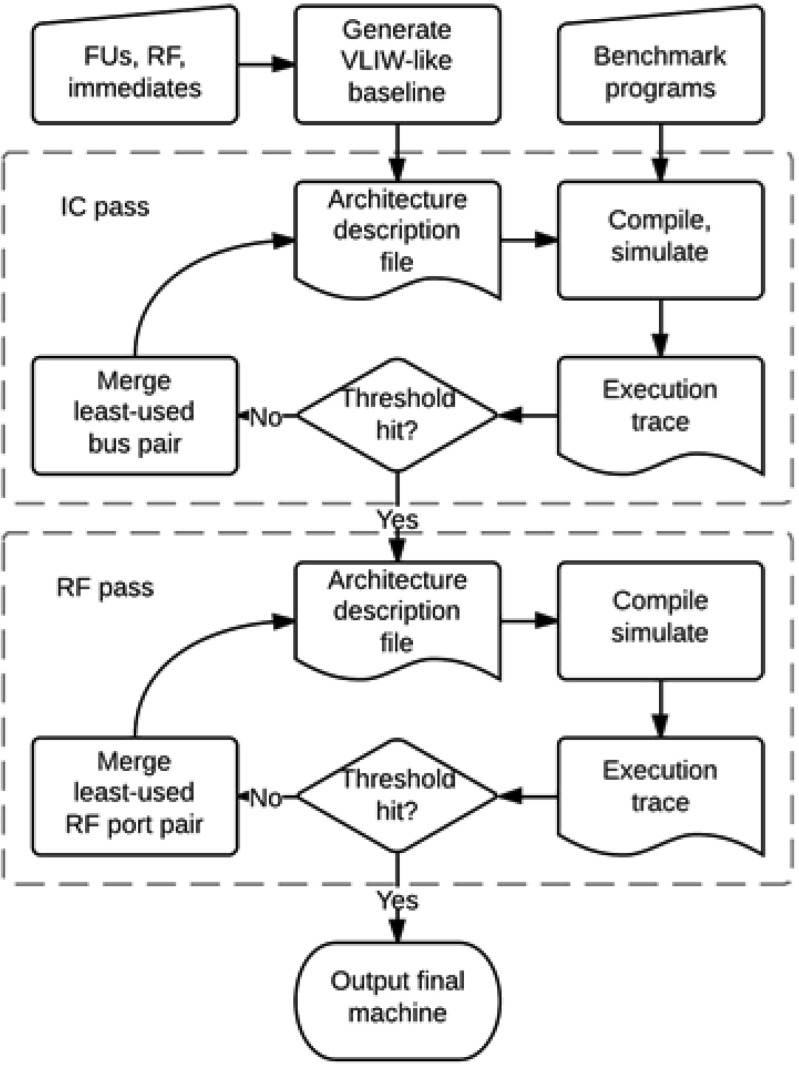
TTA resource merge flow in TCE.

TCE includes a plugin-based framework for automatic design space exploration. The framework is built around a design space database (DSDB) which stores processor configurations: plugins can then be invoked on a configuration to generate one or more modified configurations. Plugins are guided by the simulated performance of the design in benchmark programs, and by an area and power model characterized on a 130 nm ASIC technology. Two examples of plugins are GrowMachine which adds computational resources to the TTA until no more parallelism can be extracted from the benchmark programs, and ShrinkMachine which removes as many resources as possible while honouring a real-time constraint. The framework includes sufficient functionality to generate processors from scratch, but in practice a better quality is currently obtained by hand-crafting the design, while using the explorer to automate repetitive tasks. Explore has plugins designed to produce efficient TTA interconnection networks (ICs) [24]. The IC is particularly tedious to design by hand, and has a very large design space which poses difficulties for automated search. The main idea is to start from an IC topology identical to a conventional VLIW, and then shrink it through repeated bus merge and register file port merge operations. The resources to be merged are chosen by profiling the benchmark workload and generating a resource utilization covariance matrix: resources with low covariance are seldom used simultaneously and, therefore, can be merged without impacting the performance much. The resulting automated process is shown in Fig. 5.

#### Configuration of TTA Accelerators

**Figure 6:**
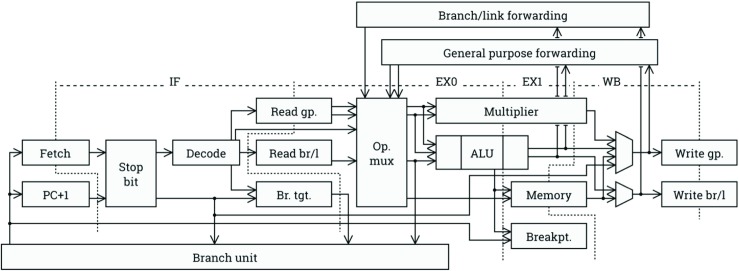
Pipeline organization of a default *ρ*-VEX datapath.

A library of components has been built for integrating TTAs with AXI interfaces, including caches and Load/Store Units which communicate over AXI. Tasks such as starting and stopping execution are done through a debug and control interface, usually accessed through an AXI slave peripheral. The interface can currently be used to clock gate an unused accelerator by placing it in a break state; it would also be straightforward to add further low-power modes. The interface also supports debug features such as multiple breakpoints, stepped execution, and examination of various performance counters and internal state data of the processor. In order to facilitate testing of ASIC prototypes with a limited number of IO pins, a JTAG interface wrapper gives access to memories and control interfaces over a four-wire interface. See Fig. 6 for the default organization of a rvex pipeline.

### Adaptable cores: *ρ*-VEX

The *ρ*-VEX is a dynamically reconfigurable VLIW processor. It is implemented in VHDL and can be synthesized to FPGA targets for prototyping. The reconfigurable concepts used in the core can also be used in ASIC technology as they do not require the reconfigurable properties of the FPGA. The VEX instruction set used in the processor is created by HP. Its target application domain is media applications (image/video). The core is design-time reconfigurable through VHDL generics in a number of properties: Issue width, functional unit layout, cache sizes, etc. Run-time reconfigurability can also be disabled to save chip area. The run-time reconfigurable core can change its issue width dynamically by splitting the core into smaller subcores that have their own execution context. This way, the processor aims to run a wide range of workload types in the most efficient way possible. Single threaded applications with a high level of parallelism in the code can run on a large high-performance core, and multi-threaded applications can run in parallel on smaller cores.

**Figure 7:**
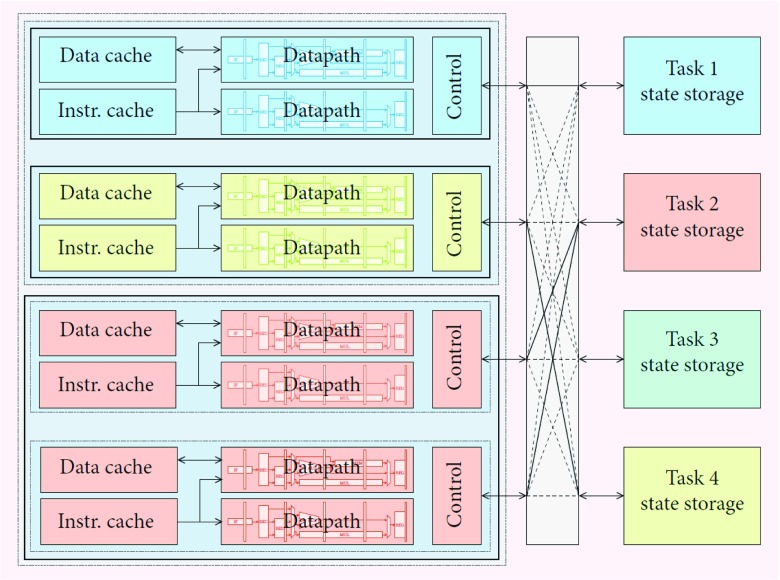
*ρ*-VEX datapaths and program state storage areas (contexts). In the depicted configuration, two pairs of datapaths are assigned to context 2, consequently it is running in 4-issue VLIW mode. The other two pairs are assigned to task 1 and 4, who are both running in 2-issue mode. Context 3 is currently disabled. Switching between configurations can be done at the cost of a pipeline flush.

The core supports a number of execution *contexts* that can be assigned to pipeline pairs (*lanepairs*). Both properties are design-time reconfigurable. When a single context is assigned to all lanes, the core runs in the widest configuration. By assigning different contexts to the lanepairs, they will each run a separate program (see Fig. 7). The *ρ*-VEX uses memory-mapped registers to control its configuration. Supervisor software can load a set of programs into the execution contexts, and use the configuration control registers to select which programs are being executed and in which configuration. The contexts contain all information needed to resume execution when being suspended (for example, when all the lanes are assigned to another context). This includes all registers, program counter, trap/interrupt state etc.

The *ρ*-VEX platform consists of the synthesizable VHDL designs of the core, cache and a number of peripherals. On the software side, there is an interface tool that can connect to the core and provides the user with extensive debugging capabilities and full control of the processor. The implementation of support for AlmaIF is discussed in Section 4.2.2. There are a number of different compilers that can target the *ρ*-VEX, there is a port of binutils and GDB, there is basic Linux support (uCLinux with a NOMMU kernel), runtime libraries (the uCLibc C standard library and newlib) and an architectural simulator.

#### Design-Time Customization

The *ρ*-VEX processor is both run-time and design-time configurable. When creating a general-purpose platform that must be able to provide high performance for single threads and high throughput for multiple threads, the full dynamic 8-issue core can be used. However, the area utilization of this configuration is high. If the workload is highly parallelizable, a static 2 or 4-issue core configuration can be used. The area utilization is considerably lower, and single-thread performance can be sacrificed because the workload will rely on multithreading to achieve high performance. The reduction in area allows more core to be placed on the chip, resulting in higher total throughput. In addition to design-time configuration of the processors, multiple *ρ*-VEX cores can be connected to each other using local scratchpad memories, preventing the need to access global memory.

#### AlmaIF Integration with pocl on *ρ*-VEX

As an example of how the AlmaIF interface may be implemented in hardware for an existing accelerator, we report the implementation for the *ρ*-VEX processor in more detail.

The *ρ*-VEX processor is a VLIW processor, with configurable amount of virtual processors, known as contexts. A control unit known as the reconfiguration controller manages the division of the available computational resources amongst these contexts. All contexts share a common instruction memory, and a common data memory. To allow the processor to be controlled externally, for debugging or otherwise, a number of control registers are available through a debug port.

This was mapped to AlmaIF as follows. First, an additional access port to the instruction and data memories was added to allow the host processor to access these through the respective AlmaIF memory regions. As the *ρ*-VEX has only one data address space, the AlmaIF parameter memory region was simply mapped to the *ρ*-VEX data memory as well, making the size of the *ρ*-VEX data memory the sum of the AlmaIF data and parameter memory sizes.

The *ρ*-VEX debug port serves a similar purpose as the AlmaIF control region. However, the *ρ*-VEX and AlmaIF registers do not map one-to-one. Therefore, a translation unit was constructed that emulates the AlmaIF registers. In order to still allow the *ρ*-VEX debug port to be used natively, it was also mapped to AlmaIF directly, using a memory region not specified by AlmaIF.

Each *ρ*-VEX context has its own set of debug registers. Therefore, each *ρ*-VEX context is represented as an AlmaIF core.

## Implementation

### Hardware

To generate a SoC platform, IPs need to be generated from the accelerator’s sources that can be used by Vivado to integrate into a AXI-based project. These steps can be performed according to the accelerator’s documentation. Note that for the accelerators that are available at the time of writing, certain levels of configuration are available when generating the IPs. The TTAs that are generated from the TCE (TTA Co-Design Environment) are fully customizable. A graphical user interface is available to accomplish this, see (http://tce.cs.tut.fi/user_manual/TCE/) for more information. The *ρ*-VEX has a number of design-time options that are available before generating the IP (by means of a configuration script), and additional options that are implemented as VHDL generics that can be set in Vivado after importing it. The options that are handled by the configuration script are for example the pipeline organization and control registers, and the VHDL generics include issue width, memory/cache sizes and performance counters.

After adding the desired IP blocks to a Vivado project, the user needs to assign address spaces according to the memory regions discussed in Section 3 and customize the clocking. This should be done considering the configuration of the accelerators, as this influences the achievable timing considerably. For instance, a 2-issue *ρ*-VEX with 7-stage pipeline and forwarding disabled can be expected to operate on much higher frequencies compared to an 8-issue configuration with forwarding and a shorter pipeline.

### Software

The AlmaIF interface was added as a target in the pocl runtime system in order to support the execution of OpenCL programs. This section discusses the way in which this can be enabled during compilation of the pocl runtime itself and how programmers can offload tasks to AlmaIF accelerators.

#### Compile-Time

The target accelerators need a compiler capable of accepting OpenCL input to generate a suitable binary. Furthermore, an OpenCL command queue mechanism must be provided to have the accelerators check for new work from the OpenCL runtime. The binary contains a field to identify the accelerator it was compiled for, so that the runtime can verify it is loading a proper binary. This field can be used to include the configuration of the accelerator (e.g., the VLIW issue width or pipeline latencies) or a hash which is more appropriate in case of the TTAs that have an enormous configuration space.

The pocl OpenCL framework was extended with an AlmaIF target that is able to drive accelerators. After compiling pocl with the interface enabled, OpenCL programs can be linked together with the pocl runtime library.

#### Run-Time

At run-time, the user needs to pass the location of the accelerators in the address space. A dedicated Linux device driver that supports bus scanning or other means of hardware detection is future work. The memory addresses can be passed to pocl by means of environment variables. When calling the program, the user needs to supply the appropriate binaries for the accelerators to allow the runtime environment to load them into the accelerator’s memories.

## Experiments/Evaluation

We use a standardized FPGA-based platform for development and demonstration purposes. This platform consists of a Zynq ZC7020 based FPGA development board with an high-quality camera connected, HDMI interfaces and an FPGA configuration with everything necessary to prototype real time video/image processing solutions at system level.

### Target platform: Xilinx Zynq

The Xilinx Zynq series of system-on-chip FPGAs consist of a hard Processing System (PS) with a dualcore Cortex A9 ARM processor, and a varying amount of Programmable Logic (PL). The main resources in the PS are:

- Dual-core ARM Cortex-A9
- L2 cache, 512KB
- On-chip memory, 256 KB
- DDR controller
- Miscellaneous peripherals (USB, Ethernet, etc.)

There are various data interfaces between the PS and the PL:

- Two 32-bit general-purpose AXI ports (GP) (PL Master)
- Two 32-bit general-purpose AXI ports (GP) (PS Master)
- Four 64-bit high-performance (HP) AXI ports (PL Master)
- One 64-bit AXI Accelerator Coherency Port (ACP) (PL Master), accesses the PS cache hierarchy.

The HP and ACP ports give better throughput than GP, so they should be used for large data transfers. GP ports should be used for programming accelerators and control traffic. Using the ACP may simplify programming of the ARM, but it can be inefficient for very large data transfers that thrash the cache and this can significantly slow down the performance of the ARM. The PS is typically clocked at ca. 667 MHz and the practical maximum of PL is in the range of 100– 150 MHz. Any IO between the PS and PL has some latency overhead due to the clockdomain crossing (CDC).

**Figure 8:**
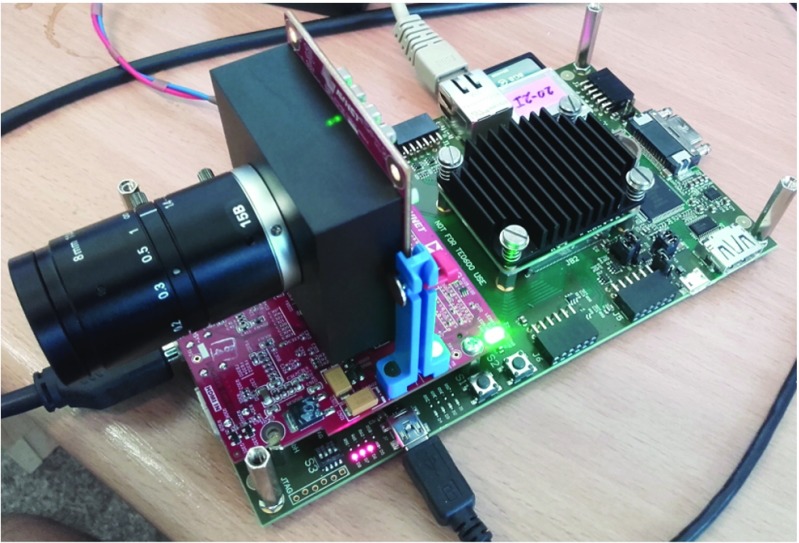
Evaluation setup consisting of a Trenz carrier board with Xilinx Z0720 SoM and Python 1300 camera on a HDMI I/O board.

**Figure 9:**
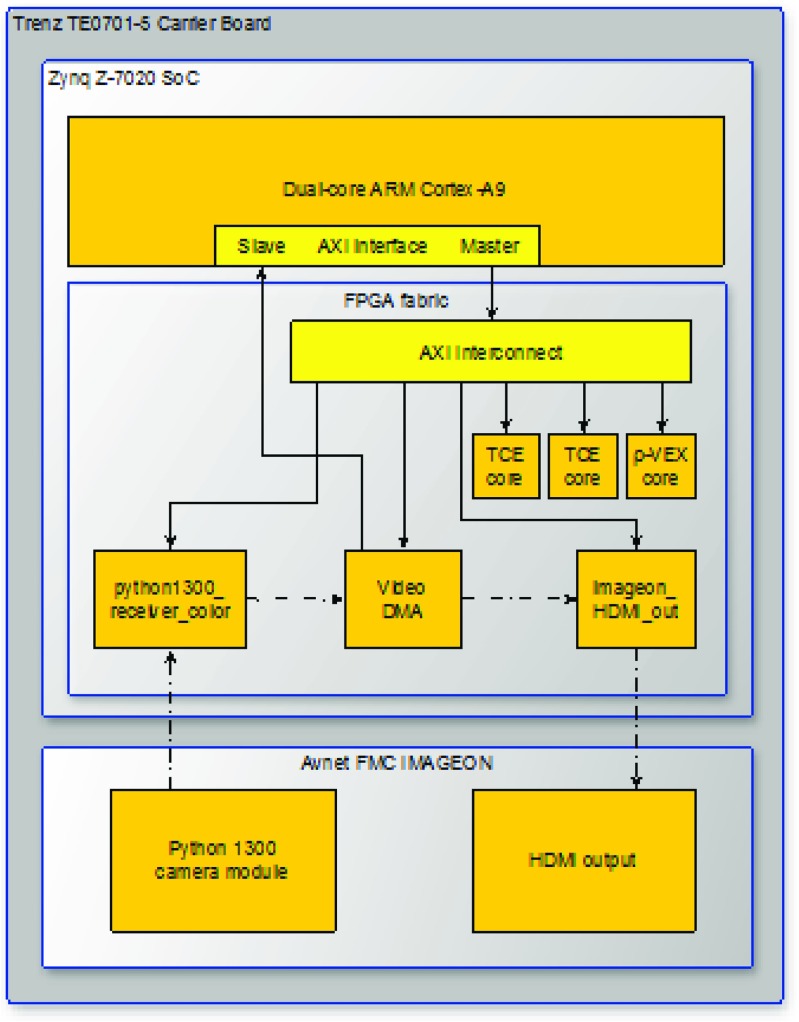
Architecture overview of the base platform with the camera and I/O components and the added accelerators.

We have expanded the prototype FPGA target board, a Trenz carrier board TE0701-5 with Zynq System on Module (SoM) TE0720-2-2if, with a Python 1300 camera module and Avnet FMC IMAGEON HDMI I/O extension board. The complete hardware setup can be seen in Figs. 8 and 9. The FPGA part of the platform contains a video chain running from Python camera input with 1280x1024p60 in 16-bit YUV422 pixel format through a video DMA unit that stores video frames to DDR memory and reads them back independently for video output by a HDMI output interface. The FPGA design of the platform is provided in the form of a Xilinx Vivado project and supports custom hardware accelerator extensions. Software support for the platform is built on Petalinux. The platform contains drivers for its hardware components located in FPGA fabric and a simple sobel filter example (implemented as C code for ARM) demonstrating how to initialize video stream using drivers and how to use video DMA to process individual frames and synchronize input and output video streams.

Detailed descriptions and an evaluation version of the platform is provided by UTIA as a downloadable package (http://sp.utia.cz/index.php?ids=results&id=apcp).

### Multi-accelerator Prototype

**Figure 10:**
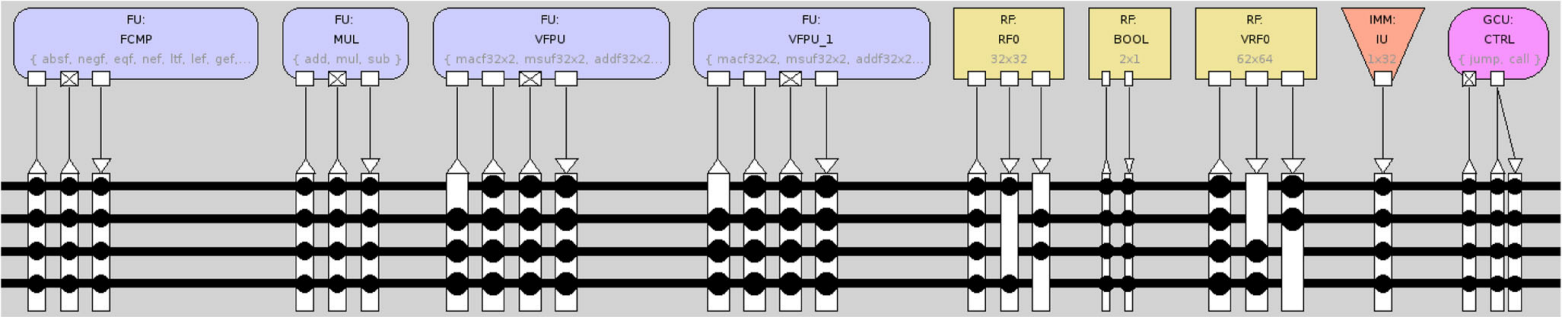
Organization of the TTA processor as used in the prototype.

For the integration prototype, an FPGA design is created with two TCE cores and one 4-issue *ρ*-VEX core, and another with two *ρ*-VEX cores. The TCE cores were clocked at 200 MHz, while the *ρ*-VEX cores were clocked at 66 MHz. Any combination of two cores can be chosen from the application software by using the standard OpenCL APIs. A Proof-of-concept multi-kernel application software was written for this platform design instance, based largely on the Sobel example. An example TCE processor design was used with two 2-cycle ALUs and a single 3-cycle 32x32b multiplier unit. The configuration can be seen in Fig. 10. The instruction, data and parameter memories have 64 kB, 32 kB and 2 kB of memory, respectively. In addition, the core has 4 kB of scratchpad memory. The organization of the processor is depicted in Fig. 10.

**Figure 11:**
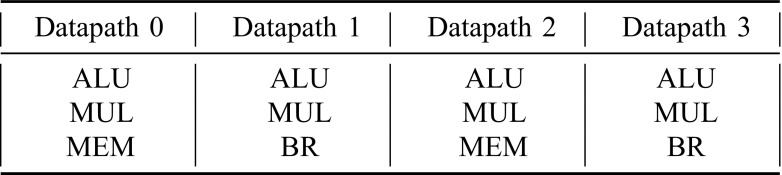
Functional units in the pipelines of the *ρ*-VEX as used in the prototype.

The default *ρ*-VEX configuration used in the ALMAIF platform is a 4-issue reconfigurable core that can split into a 2 core (2-issue). It has 2 hardware contexts (virtual processors) that can hold a full program state. This way, the 2 cores can each run a process independently. The core supports variable-length instruction words to improve the encoding efficiency of the binary code, which results in smaller binaries. The data path consists of 4 ALUs, 4 16x32 multiplication units, 2 MEM (Load/Store) and 2 branch units, depicted in Fig. 11. As discussed earlier, the default size is 32 kB for instruction, data, and parameter memory. The memories have 2 access ports. This is needed in order to provide 1 port per context when running in split 2-issue mode, but it also supports multiple Load/Store operations when running in merged 4-issue mode. The core has full debug functionality including 4 hardware breakpoints and a set of 32-bit performance counters per context. When using *ρ*-VEX as accelerator, as is the most common use case in this platform, these options may be disabled after debugging and optimizing the application as this will save considerable resources and improve timing.

FPGA utilization for the 3-core design is shown in Table 3. The last row shows the total utilization of the FPGA chip’s resources.

**Table 3:** Area utilization of the various components in the prototype platform.

Name	LUTs	Registers	BRAM	DSP
*ρ*-VEX core	13052	6818	41	8
TCE core 0	2873	2246	17.5	3
TCE core 1	2898	2247	17.5	3
Python1300	6695	8332	18	12
VDMA	3760	6430	8.5	0
HDMI	993	2344	2.5	0
Others	2989	5341	0	0
Total	33260	33758	105	26
	(62%)	(32%)	(75%)	(12%)

**Figure 12:**
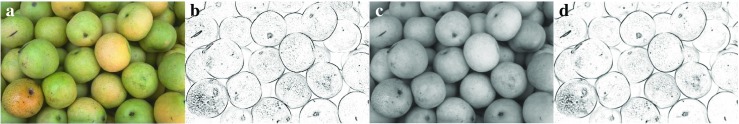
From left to right: **a** Original image, **b** Sobel filter applied **c** Blur filter applied, and **d** both filters applied.

The example application applies a Sobel filter and a 3x3 box blur filter, in that order, to the image acquired from the camera, and pushes the result to the HDMI output. The filtering is executed on two AlmaIF devices, each handling one filter at the time. The host application handles partitioning the work into smaller segments and enqueuing kernel execution and the required data transfer operations in each device’s command queue. The queues are then executed by the pocl runtime, with data dependencies between the two queues resolved by event waitlists. An example of intermediate results and the final result can be seen in Fig. 12.

Although the prototype was made only for proving that the integrated hardware and software stack works, evaluation numbers were produced as a base line for further optimization. For a 1280-by-1024 image, the runtimes for the AlmaIF-based ALMARVI platform instance with different kernels assigned to different device types are depicted in Table 4.

**Table 4:** Performance of the two available AlmaIF accelerators on the image processing filters.

Accelerator					
Sobel	Blur	Read	Write	Sobel	Blur
		(ms)	(ms)	(ms)	(ms)
TCE	TCE	124	146	967	455
TCE	*ρ*-VEX	261	300	967	2899
*ρ*-VEX	TCE	262	301	757	457
*ρ*-VEX	*ρ*-VEX	399	455	757	2898

## Conclusions

The increasing demand for higher performance at lower energy budgets, combined with the decreasing improvements from technology scaling, leads to increasingly heterogeneous systems where tasks are executed on custom-designed accelerators.

In this context, we implemented an OpenCL platform capable of utilizing multiple different accelerators together in a single program. The pocl OpenCL framework is extended with a target that can drive these accelerators. Because of the generic accelerator interface, the same pocl runtime can be used for any current and future accelerator that adheres to the specification. At run-time, the user can choose which accelerators to enable and the distribution of the kernels to the different accelerators. In the future, this distribution can also be performed by the system based on various metrics such as task characteristics or the system’s current power policy.

To the programmer, this fully heterogeneous platform is fully transparent, by making use of the OpenCL framework and compiling for the different accelerators through different compiler back-ends.
